# Scalp microbiome of healthy women wearing hijab compared to those not wearing hijab: a cross-sectional study

**DOI:** 10.1038/s41598-023-38903-2

**Published:** 2023-07-21

**Authors:** Sandra Widaty, Lis Surachmiati, Lili Legiawati, Sondang Pandjaitan Sirait, Inge Ade Krisanti, Windy Keumala Budianti, Eliza Miranda, Rahadi Rihatmadja, Caroline Oktarina

**Affiliations:** Department of Dermatology and Venereology, Faculty of Medicine Universitas Indonesia – Dr. Cipto Mangunkusumo National Central General Hospital, Jakarta, 10430 Indonesia

**Keywords:** Bacteria, Microbial communities, Fungi, Microbial genetics

## Abstract

Use of hijab can influence the scalp’s condition, including its microbiome. To date there has been no study comparing scalp microbiome in women wearing hijab to that in women not wearing hijab. This was a cross-sectional study conducted from August 2019 to April 2021. Healthy women aged 18 years old or older who had not undergone menopause were recruited. Those in the hijab group should wear hijab minimum 8 h a day for at least 5 years. After wash-out period, the sample was collected from the subject’s scalp. Next Generation Sequencing (NGS) was performed with primer V3-V4 region of 16S rRNA and ITS1 DNA for bacteria and fungi, respectively. Alpha diversity and beta diversity were identified, along with functional analysis. Actinobacteria and Ascomycota were the most dominant phyla on the scalp. *S. capitis* was more prominent in the hijab group while *S. cohnii* was more prominent in non-hijab group. Additionally, *M. restricta* was more common in hijab group while *M. globosa* was more common in non-hijab group. This study emphasizes the difference of scalp microbiome in women wearing hijab compared to women not wearing hijab, which indicated that women wearing hijab are more prone to seborrheic dermatitis.

## Introduction

The scalp skin is inhabited by abundant microorganisms, known as microbiota. The microbiota has a mutual relationship with the host as it plays a role in skin inflammatory responses and homeostasis. The equilibrium of microbiota-host relationship is necessary to keep the skin vigorous. The skin microbiota develops over the years with different dominant phyla each life stage. Also, different sites have different microbial communities. Multiple factors influence the skin microbiome, some of them are still unknown^1^. Gender, age, hair treatment, lifestyle, and geographical regions are several factors that are thought to influence the scalp microbiome^2,3^.

Dysbiosis of the scalp microbiome might influence the course of some disorders e.g., seborrheic dermatitis, androgenetic alopecia, alopecia areata, syphilitic alopecia, cicatricial alopecia, folliculitis decalvans, folliculitis, and tinea capitis^3,4^. In addition, dysbiosis of scalp microbiome was also reported in sensitive scalp which was characterized by various abnormal sensory symptoms (pruritus, pain, tingling, burning)^5^. Both fungal and bacterial communities in the scalp can interact with the host, leading to alteration of the scalp health. Hence, the scalp microbiome should also be given attention when managing scalp diseases^3^.

Indonesia is a tropical country in which majority of its citizens were Muslim. Most of the Muslim women have practice the use of hijab since they were a child. A hijab is worn to cover the hair and body with various styles^6^. The use of hijab, especially for a long time, might occlude the scalp which results in accumulation of sebum and scalp. These can predispose the women to itch and dandruff. In addition, some of the women who do not dry their hair and use hijab directly might cause the hair flat and damaged. Use of hijab can also reduce sunlight exposure to the scalp which reduces the vitamin D source^7^. A previous study also showed that use of hijab was associated with increased transepidermal water loss (TEWL) of the scalp^8^. Considering these changes, this study aims to compare the scalp microbiome of healthy women wearing hijab to those not wearing hijab.

## Results

**Figure 1:**
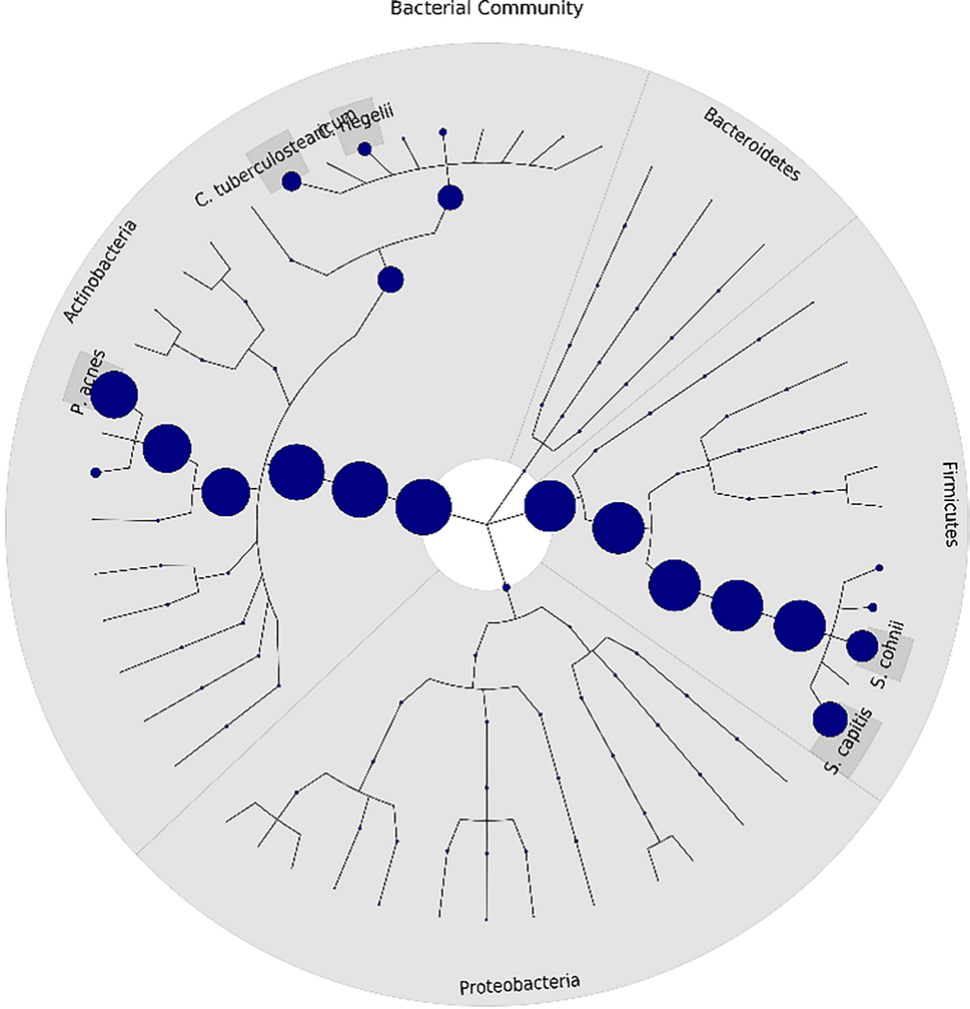
Bacterial taxonomic tree of all subjects (generated using GraPhlAn tools from https://huttenhower.sph.harvard.edu/graphlan/).

**Figure 2:**
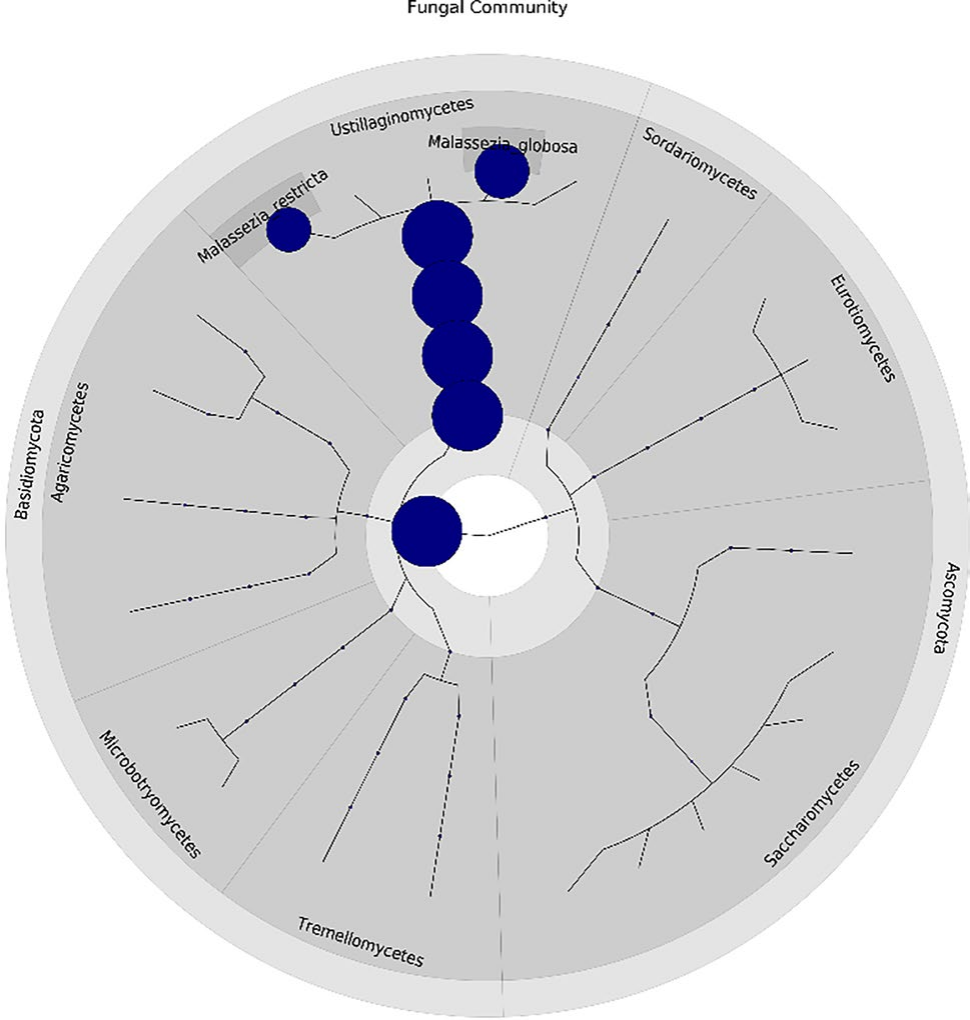
Fungal taxonomic tree of all subjects (generated using GraPhlAn tools from https://huttenhower.sph.harvard.edu/graphlan/).

A total of 96 subjects were recruited to the study, comprising 48 healthy women wearing hijab and 48 healthy women not wearing hijab (Supplementary Fig. S1). Of all bacteria identified, the most abundance bacterial phyla on the scalp were Actinobacteria, Proteobacteria, Firmicutes, and Bacteroidetes (Fig. 1). The three most dominant species were *Propionibacterium acnes, Staphylococcus capitis,* and *Staphylococcus cohnii.* While the most abundance fungal phyla on the scalp were Ascomycota and Basidiomycota (Fig. 2). The most dominant species were *Malassezia globosa* and *M. restricta.* Post-hoc power analysis on the fungal findings showed 99.9% power while post-hoc power analysis on the bacterial findings showed 5.3% power, indicating lack of power for the bacterial study.

**Figure 3:**
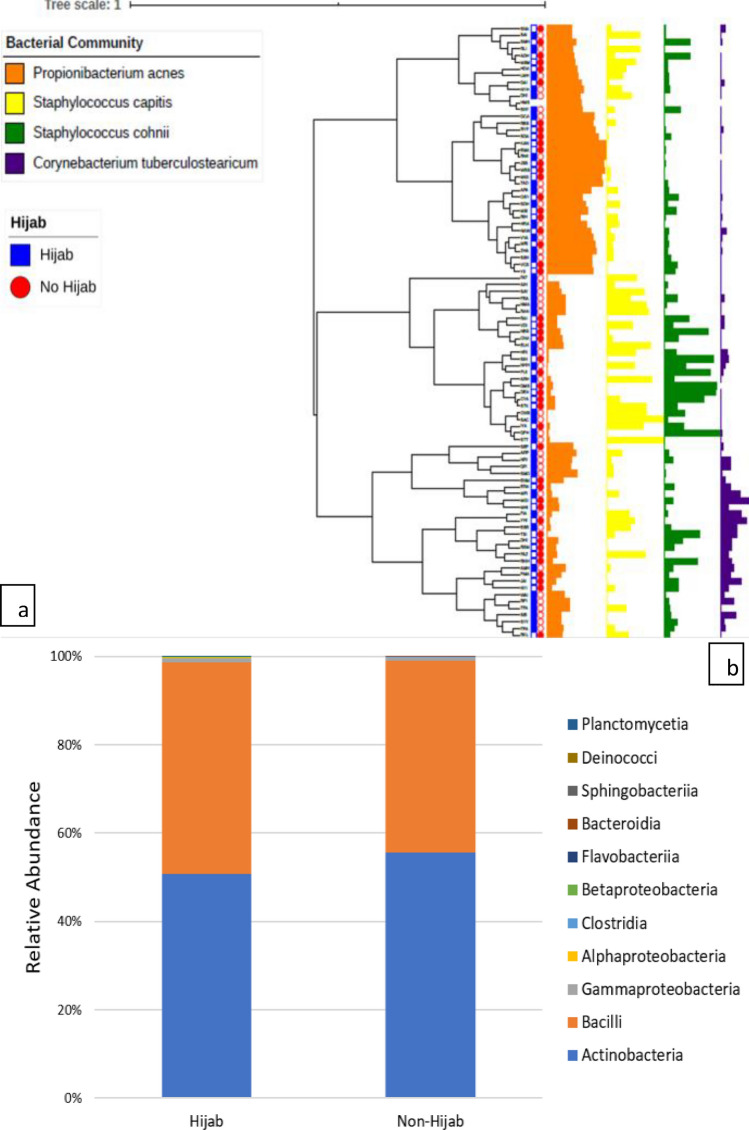
Comparison of bacterial species on scalp microbiome of healthy woman wearing hijab and not wearing hijab (generated using iTOL tools from https://itol.embl.de/ and Microsoft Excel).

**Figure 4:**
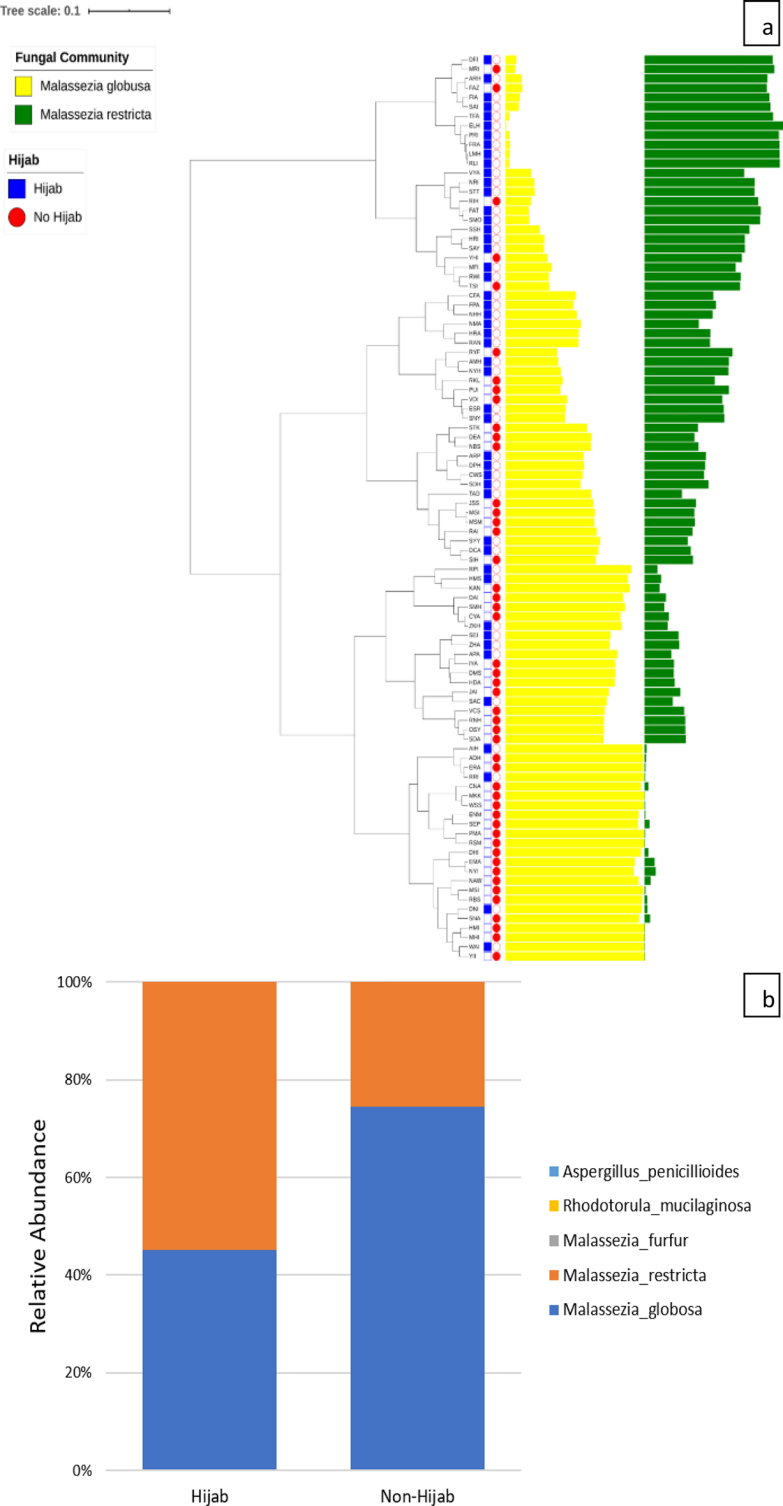
Comparison of fungal species on scalp microbiome of healthy woman wearing hijab and not wearing hijab (generated using iTOL tools from https://itol.embl.de/ and Microsoft Excel).

Beta diversity of both groups showed different microbial dominance (Figs. 3 and 4). Both groups showed *P. acnes* dominance. However, *S. capitis* was more prominent in hijab group while *S. cohnii* was more prominent in non-hijab group. Additionally, *M. restricta* was more common in hijab group while *M. globosa* was more common in non-hijab group.

**Figure 5:**
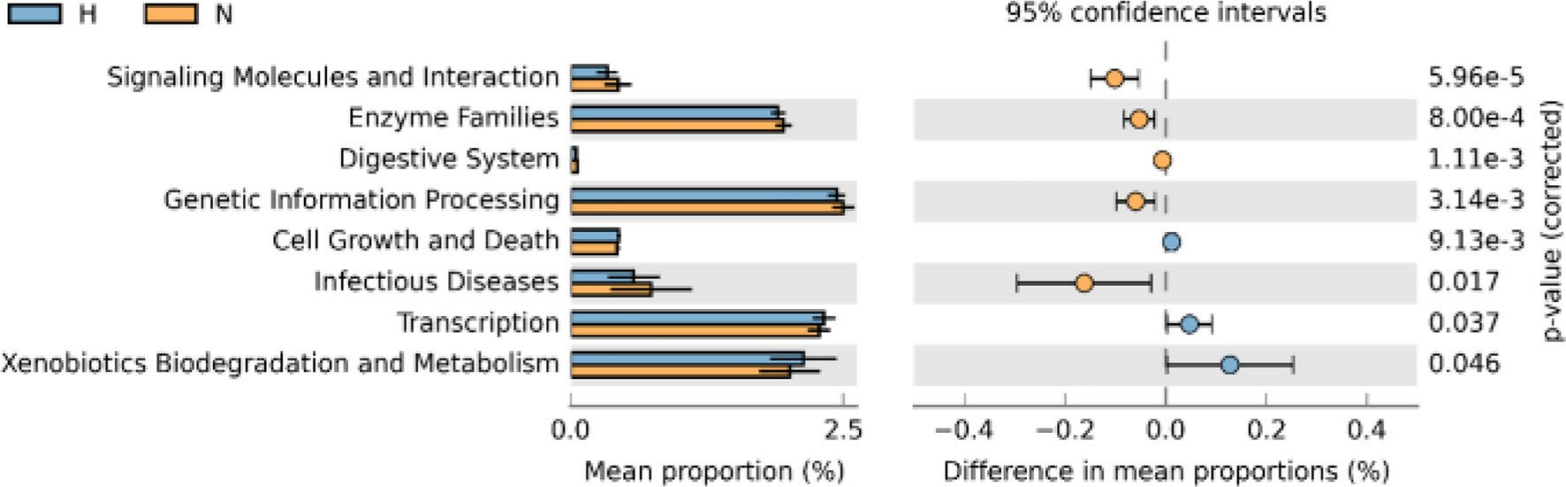
Functional analysis of KEGG pathway L2 of scalp microbiome in hijab group vs. non-hijab group (generated using STAMP tools from https://beikolab.cs.dal.ca/software/STAMP).

**Figure 6:**
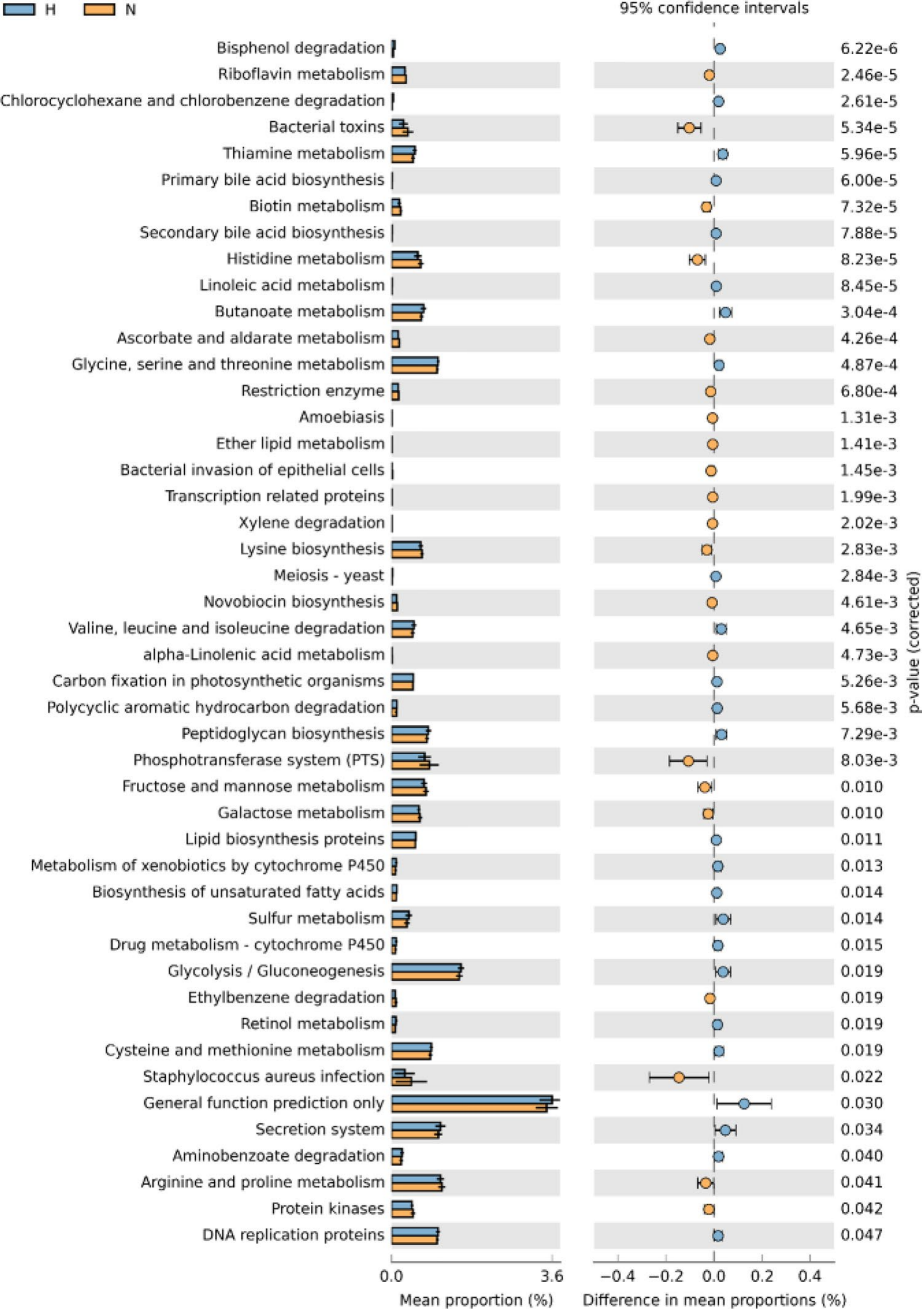
Functional analysis of KEGG pathway L3 of scalp microbiome in hijab group vs. non-hijab group (generated using STAMP tools from https://beikolab.cs.dal.ca/software/STAMP).

Further functional analysis is shown in Figs. 5 and 6. According to KEGG pathway L2 analysis, the hijab group had statistically significant lower signaling molecules and interaction, enzyme families, digestive system, genetic information processing, and infectious diseases compared to non-hijab group. The non-hijab group had statistically significant lower cell growth and death, transcription, as well as xenobiotics degradation and metabolism compared to hijab group. According to KEGG pathway L3 analysis, the hijab group had statistically significant lower riboflavin metabolism, bacterial toxins, biotin metabolism, histidine metabolism, ascorbate and aldarate metabolism, restriction enzyme, amoebiasis, ether lipid metabolism, bacterial invasion of epithelial cells, transcription-related proteins, xylene degradation, lysine biosynthesis, novobiocin biosynthesis, alpha-linoleic acid metabolism, phosphotransferase system, fructose and mannose metabolism, galactose metabolism, ethylbenzene degradation, *S. aureus* infection, arginine and proline metabolism, as well as protein kinases compared to non-hijab group. On the other hand, the non-hijab group had significantly lower bisphenol degradation, chlorocyclohexane and chlorobenzene degradation, thiamine metabolism, primary bile acid biosynthesis, secondary bile acid biosynthesis, linoleic acid metabolism, butanoate metabolism, glycine, serine, and threonine metabolism, meiosis, valine, leucine, and isoleucine degradation, carbon fixation in photosynthetic organisms, polycyclic aromatic hydrocarbon degradation, peptidoglycan biosynthesis, lipid biosynthesis proteins, xenobiotics metabolism by cytochrome P450, biosynthesis of unsaturated fatty acids, sulfur metabolism, drug metabolism, glycolysis, retinol metabolism, cysteine and methionine metabolism, general function prediction, secretion system, aminobenzoate degradation, and DNA replication proteins.

## Discussion

This study showed that Actinobacteria, Proteobacteria, Firmicutes, and Bacteroidetes were the dominant phyla in the scalp of healthy women. Similarly, Watanabe et al. reported that Actinobacteria, Proteobacteria, and Firmicutes were the dominant phyla in the scalp of 109 healthy males and females^2^. Actinobacteria, Firmicutes, and Proteobacteria are also the most dominant phyla in hair follicle^4^.

The three most dominant species were *Propionibacterium acnes, Staphylococcus capitis,* and *Staphylococcus cohnii.* Lousada et al. also reported that *Propionibacteria, Staphylococci,* and *Pseudomonades* were the dominant bacteria in the hair follicle^4^. Watanabe et al. also reported similar bacteria on the scalp hair shaft of healthy individuals^2^. These bacteria are found to be abundant on the vertex and occipital regions of the scalp. *Propionibacteria* is also predominant on the facial skin and axilla while *Staphylococcus* is predominant in axilla. Both species are associated with acne vulgaris and alopecia areata^4^.

Beta diversity of both groups showed different microbial dominance. Both groups showed *P. acnes* dominance. *Propionibacterium* resides in the hair follicle which is difficult to reach by disinfectant. Hence, its abundance is relatively constant^4^. *P. acnes* has the ability to hydrolyze triglycerides into free fatty acids. It also amplifies bacterial adherence and eliminates other strains with bacteriocins. It was also reported to increase significantly along with perturbed scalp microbiome in sensitive scalp. Sensitive scalp is characterized by pruritus, discomfort, tingling, tightness, and burning when the scalp is exposed to environment^5^. This was reported by women who used hijab^7^. From our findings, we can see that the dominant *P. acnes* species in scalp, might predispose the healthy women into sensitive scalp in both those wearing hijab and not wearing hijab.

However, *S. capitis* was more prominent in hijab group while *S. cohnii* was more prominent in non-hijab group. *S. capitis* is one of the coagulase-negative staphylococci which normally resides on the human mucous membranes and skin^9^. A previous study had isolated *S. capitis* from the foot’s skin of healthy subjects. *S. capitis* was proven to be beneficial by producing antimicrobial peptides, which was particularly effective on eliminating Gram positive organisms, such as *S. aureus*, *P. acnes, Micrococcus luteus*, and *Bacillus subtilis*. The produced peptides were proven not to be harmful towards human keratinocytes^10,11^. However, another study reported that *S. capitis* was linked to bloodstream infections. The pathogenesis of *S. capitis* infection is thought to be multifactorial, attributed to its ability to invade and destruct epithelial cells. Similarly, *S. cohnii* is a member of coagulase-negative staphylococci which has been linked to infections^9^. *S. cohnii* has two main subspecies, which are *S. cohnii* subsp. *urealyticus* and *S. cohnii* subsp. *cohnii.* While *S. cohnii* subsp. *urealyticus* is often identified in people who stay at home, *S. cohnii* subsp. *cohnii* is often identified in hospital staffs and hospitalized patients^12^. In spite of this finding, the latest study showed that *S. cohnii* might be beneficial by managing inflammation without affecting other microbiota^13^. With this finding, we hypothesize that inflammatory skin diseases, such as seborrheic dermatitis, are more often found in women wearing hijab compared to women not wearing hijab due to the more dominant *S. cohnii* findings in women not wearing hijab. On the other hand, bacterial skin infections might be more common in women not wearing hijab compared to women wearing hijab due to the more abundant *S. capitis* in women wearing hijab.

The most abundant fungal phyla on the scalp were Ascomycota and Basidiomycota. Previous study also reported that Ascomycota was dominant on scalp of healthy subjects while Basidiomycota was more common on scalp of subjects with dandruff^14^. The most dominant species were *M. globosa* and *M. restricta.* These species are predominant in the hair follicle. *Malassezia* is associated with seborrheic dermatitis, psoriasis, and androgenetic alopecia^4^. Park et al. reported that *Malassezia* spp. was identified 0.07% of the healthy scalps and higher (2%) in the scalps with dandruff. It was also stated that the most common fungal species identified in normal scalp were *Acremonium* spp., *Didymella* spp., and *Cryptococcus* spp^14^. Different geographical area, hair care, and use of hijab can influence these different findings in our study.

It was found that *M. restricta* was more common in hijab group while *M. globosa* was more common in non-hijab group. Increased abundance of *M. restricta* accompanied by decreased abundance of *M. globosa* was reported in seborrheic dermatitis patients^4^. However, another study reported that *M. globosa* was present in 52% of seborrheic dermatitis patients’ scalp in Indonesia^15^. *Malassezia* is known to produce triglyceride lipases which cleaves the triglycerides into free fatty acids. These free fatty acids will induce inflammation on the scalp and disrupt the skin barrier of the scalp. Presence of *Staphylococcus* can aid *Malassezia* in developing dandruff and seborrheic dermatitis through sebum hydrolyzation, which supplies nutrients for *Malassezia*^3,4^. Nevertheless, the occurrence of seborrheic dermatitis is influenced by various factors, not only the role of *Malassezia*^3^. Therefore, our study suggest that both women wearing hijab and not wearing hijab might be prone to seborrheic dermatitis due to the abundant *Malassezia* findings.

This study also conducted functional analysis with Kyoto Encyclopedia of Genes and Genomes (KEGG) Pathway (www.kegg.jp/kegg/kegg1.html). It focuses on 8 KEGG pathways level 2 and 46 KEGG pathways level 3. The results suggested that infectious diseases and *S. aureus* infection were more common in the non-hijab group. Several other pathways also had significant differences between both groups. However, these results should be further studied since this is the first study in Indonesia which compared the scalp microbiome in women wearing hijab to women not wearing hijab. Limitations of this study were small sample size and use only one primer for each fungal and bacterial analysis, so that a few organisms could not be identified. In addition, the analysis on the association between hijab materials and scalp microbiome was not conducted due to insufficient data.

This study emphasizes the difference of scalp microbiome in women wearing hijab compared to women not wearing hijab. Women wearing hijab were found to have more *S. capitis* and *M. restricta* on their scalps while women not wearing hijab were found to have more *S. cohnii* and *M. globosa* on their scalps. These findings indicated that women wearing hijab are more prone to seborrheic dermatitis compared to women not wearing hijab while women not wearing hijab are more prone to bacterial skin infections. Further studies are warranted to analyze the correlation between these microbiome findings with clinical and histopathological findings as well as to search the best method to improve the scalp microbiome in women wearing hijab.

## Methods

### Study design and ethical approval

This study was conducted with cross-sectional design from August 2019 to April 2021. Samples were collected from November 2019 to March 2020 during the dry season. This was a part of larger study analyzing the characteristics of scalp microbiome and clinical features in women wearing hijab versus women not wearing hijab. The study has been approved by ethics committee of Faculty of Medicine Universitas Indonesia (ethical approval number KET-888/UN2.F1/ETIK/PPM.00.02/2019). The study protocol has been registered in clinicaltrials.gov (NCT05267119). The investigation was performed in accordance with the Declaration of Helsinki.

### Study criteria

Inclusion criteria were: (1) healthy women aged 18 years old or older who had not undergone menopause, (2) wearing hijab for at least 5 years with duration of use minimum 8 hours a day for the hijab group, and (3) providing consent to participate in the study. Exclusion criteria were: (1) had history of scalp or hair disorders (e.g. alopecia, trichotillomania, malignancy, infection), (2) currently using topical or systemic medications for hair and/or scalp, (3) currently using cytostatic agents for malignancy or other disorders, (4) had history of hypersensitivity towards shampoo’s basic ingredients, (5) pregnancy or breastfeeding, (6) currently using hair or scalp care products, (7) consuming vitamin D supplements for the past 6 months, (8) receiving topical vitamin D analogue therapy, and (9) had history of diabetes mellitus, liver disorders, renal disorders, vitiligo, and/or autoimmune diseases. All participants were asked to sign the informed consent form prior to the study. Subjects were recruited consecutively.

### Study preparation

All participants were screened first and given instructions prior to the sample collection. Each participant received 1 bottle of regular shampoo (30 ml) to be used twice a week for 2 weeks. The last hair wash was 3 days before the sample collection.

### Sample collection

Sample was collected in a sterile room. The room temperature was kept at 20 °C. The investigators used personal protective equipment (PPE), comprising surgical cap, surgical mask, disposable gloves, and disposable gown. A DNA/RNA^TM^ shield collection tube with swab was used to collect the sample. The investigator used a comb to reveal an area of 4 × 4 cm at the vertex. The area was marked. The swab was moistened with buffer solution. At each vertical, horizontal, left diagonal, and right diagonal direction, the premoistened swab was stroked 5 times. Each time comprised 10 motions back and forth. The sample was stored within the tube containing buffer solution. All samples were stored in a freezer at – 80 °C.

### DNA extraction and sequencing

Dnaeasy^®^ Powersoil^®^ Pro Kit was used for DNA extraction. The process was based on the manufacturer’s handbook. The samples were homogenized with Vortex-Genie^®^ 2. Following cell lysis, the supernatant was washed. Deoxyribonucleic acid (DNA) was then extracted. The extracted DNA was purified and precipitated. Following quality control, the DNA underwent sequencing with Illumina^®^ MiSeq^®^ Next Generation Sequencer. The primers were V3-V4 region of 16S rRNA and ITS1 DNA for bacteria and fungi, respectively. The whole sequencing process was performed based on the manufacturer’s protocol.

### Bioinformatic analysis

**Figure 7:**
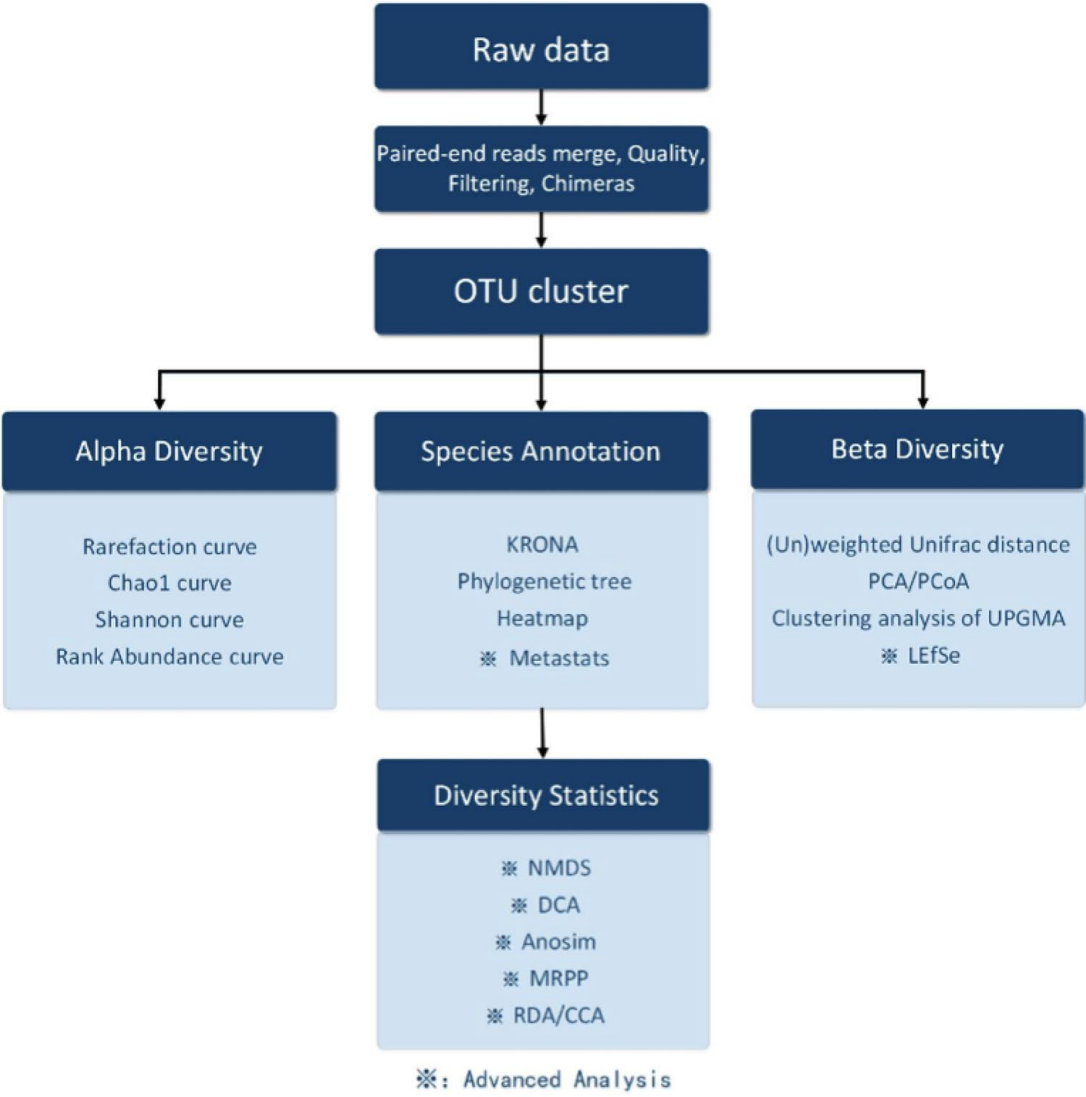
Workflow of the bioinformatic analysis.

The workflow of the bioinformatic analysis is shown in Fig. 7. We used FLASH v1.2.7 for paired end reads merger. Primer sequences were truncated. USEARCH package was used to filter the reads, calculate the abundance value, cluster operational taxonomic unit (OTU), and filter chimera. We referred to 16s sequence databases from Ribosomal Database Project for annotation. Alpha and beta diversities were analyzed with USEARCH package. Principal Coordinate Analysis (PCoA) was performed with R software (Version 2.15.3).

## Supplementary Information

Supplementary Figure S1.

## Data Availability

The datasets used and/or analyzed during the current study available from the corresponding author on reasonable request.
