# Supplementary figures and images for: Scalp microbiome of healthy women wearing hijab compared to those not wearing hijab: a cross-sectional study
Source: Sci Rep. 2023 Jul 21;13:11797. doi: 10.1038/s41598-023-38903-2 (PMC10361994; doi:10.1038/s41598-023-38903-2)

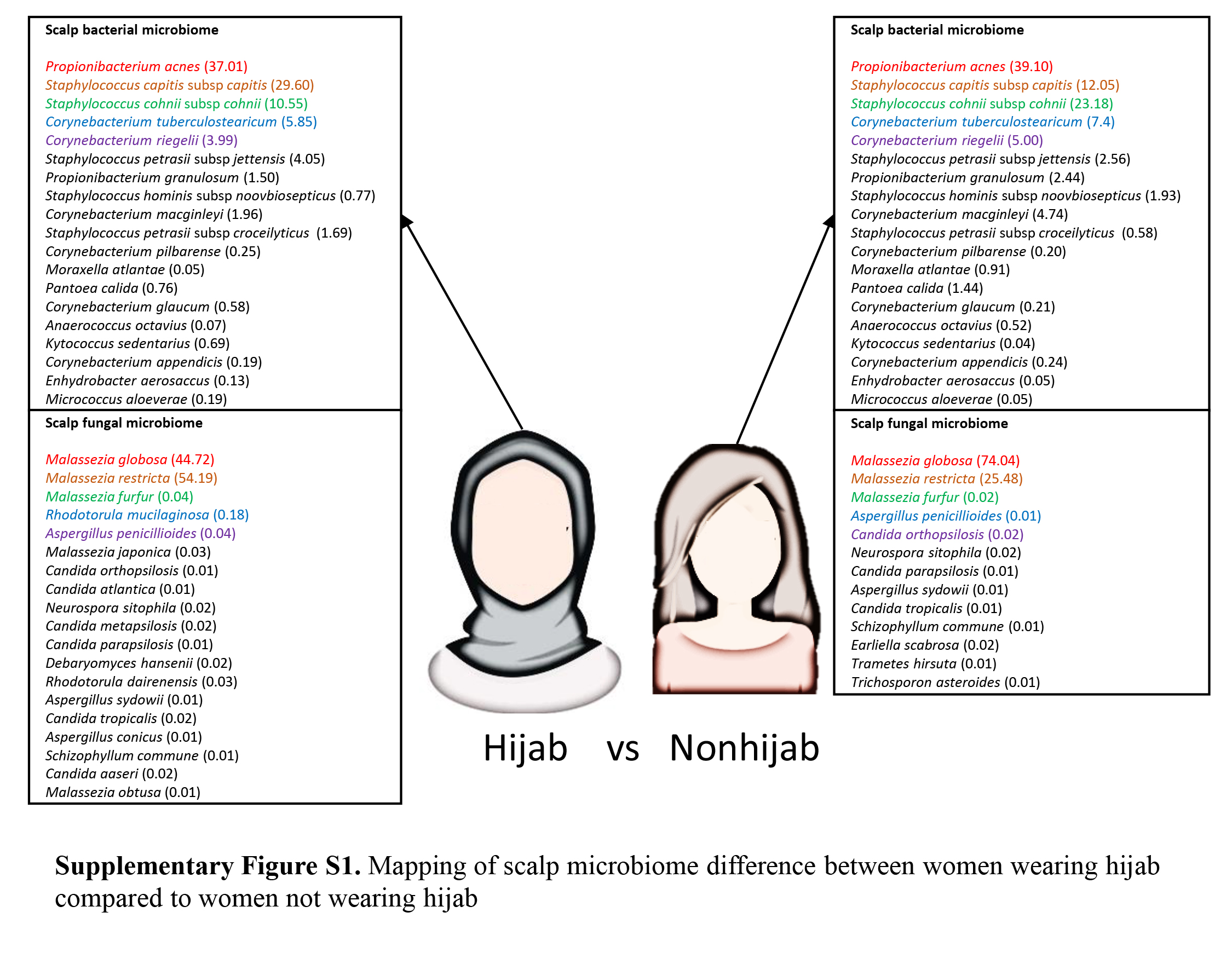

Supplement: Supplementary file 1 — Supplementary Figure S1. [file 41598_2023_38903_MOESM1_ESM.jpg]
